# Evidence-Based Medicine and the Potential for Inclusion of Non-Biomedical Health Systems: The Case for Taijiquan

**DOI:** 10.3389/fsoc.2020.618167

**Published:** 2021-01-27

**Authors:** Mark J. Langweiler

**Affiliations:** School of Health and Social Care, London South Bank University, London, United Kingdom

**Keywords:** Taijiquan, evidence-based medicine, inclusion, accreditation, authenticity, validation, evidence

## Abstract

Many traditional and complementary and alternative healthcare systems or practices, such as Traditional Chinese Medicine, taijiquan, or acupuncture, are easily found in many North American and European cities. For the most part these practices are not accredited, and their validation remains limited. This is primarily the result of the lack of modern scientific research. Additionally, the studies that are performed rely on evidence and research designs that often negate the true features of these practices with a loss of authenticity. Is it possible or even desirable for these systems to acquire accreditation and inclusion? If so, given the apparent, subjective nature of these practices, can a pluralistic approach to healthcare that retains the Western values of science and medicine be developed that yet respects the diversity of different concepts about life, health and services while permitting these practices to maintain their authenticity? And is it possible to develop a regulatory framework that practitioners can use? The current paper examines questions concerning the uses of non-Western healthcare practices without the loss of their authentic nature. The process of integration is here examined using the inclusion of taijiquan as a health-promoting martial art as the model.

## Introduction

“No attempt to refine our present medical system will prove ultimately successful unless they address the deficiencies of the most basic assumptions on which the system rests.”

-Larry Dossey

Space, Time and Medicine, 1982

The advent of evidence-based healthcare has provided us with a means to determine the effectiveness and efficacy of much of the currently relied upon medical research. However, limits of this basis for healthcare have become more evident with passing time.

Additionally, with globalization, the availability of non-traditional (by Western standards) health systems such as Traditional Chinese Medicine, taijiquan or acupuncture, are easily found in many North American and European cities. A systematic review published in The Bulletin of the World Health Organization revealed that the use of traditional and complementary/alternative therapies has been increasing worldwide (WHO, 2013). The reasons are varied. For example, patients at the Royal London Hospital for Integrated Medicine, when questioned, stated that they attended due the failure of other treatments, personal or cultural preferences or because they experienced adverse side effects with biomedical treatments (Sharples et al., 2003). In many instances, patients turn to complementary or alternative practices as a last resort, a “last hope,” for serious diseases such as cancer (Verhoef et al., 2005). Yet for the most part, these nonstandard practices are not accredited by the accrediting agencies, and their Western research validation remains limited. This is primarily the result of the lack of modern scientific research or clarity about what constitutes evidence in the modern scientific sense. However, is it possible that this mode of understanding used in conjunction with practices such as taijiquan, for example, is not a viable way to understand the effectiveness of these practices? Further, the studies that are performed rely on research designs that often negate the true features of these practices with an attendant loss of authenticity. Is it possible or even desirable for these practices to acquire accreditation? If so, given the often apparent subjective nature of these practices, can a pluralistic approach to healthcare that retains the Western values of science and medicine and yet respects the diversity of different concepts about life and health be developed while permitting these practices to maintain their authenticity? And, is the development of a regulatory framework that practitioners can use even possible? The current paper will examine these and related questions concerning the uses of non-Western healthcare practices, their accreditation and the possibility for incorporation into today’s healthcare systems without the loss of their authentic nature. Using the inclusion of taijiquan as a health-promoting martial art as the model, the process of integration will be examined from a clinical aspect, as well as the research methodology used to potentially validate its inclusion.

**FIGURE 1:**
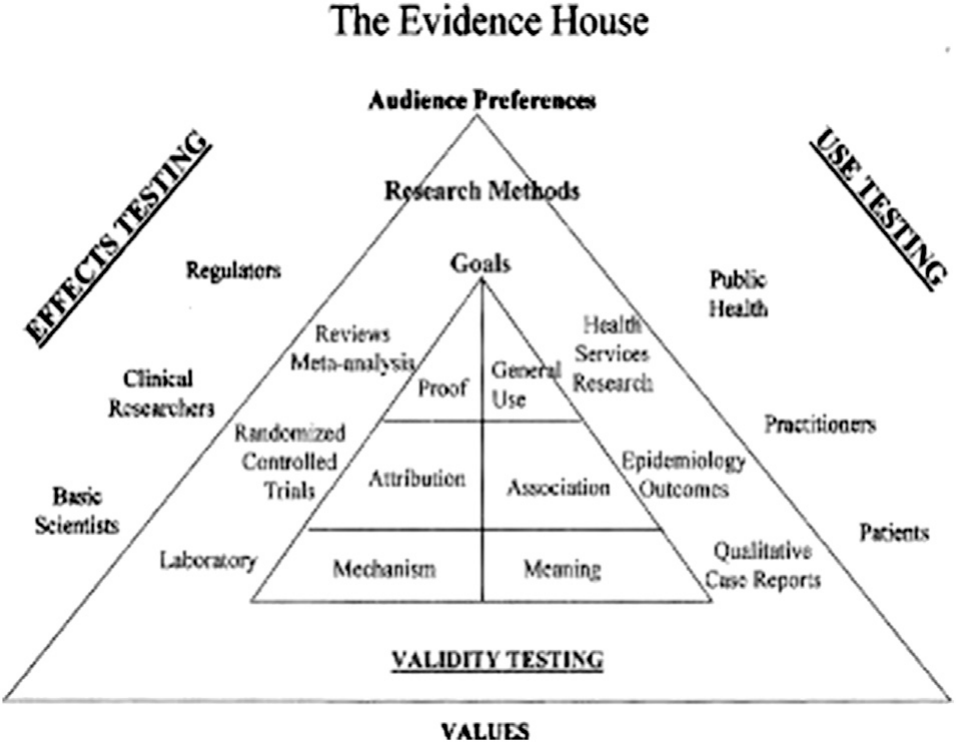
The evidence house. (“Evidence, Ethics and the Evaluation of Global Medicine.” From The Role of Complementary and Alternative Medicine: Accommodating Pluralism, Daniel Callahan, Editor, pp. 137–137. Reprinted with permission. www.press.georgetown.edu).

## Evidence and Evidence-Based Care

Before looking at evidence-based care, it is important to gain an understanding of what constitutes evidence and how this may be related to the development of the knowledge that enters the pantheon of clinical care. Over the last several decades and within the general scientific community, especially within medical research, evidence tends to be conceived as a neutral factor based on what is observable and useable. The idea that within the Western research paradigm, to use the term first coined by Thomas Kuhn [Kuhn, 1962 (1970)] to describe a set of specific frames of reference that depend on theoretical assumptions and presuppositions, is devoid of meaning and an objective view of reality has come under increasing skepticism. Developing out of the idea that our understanding is based on context, feminist theorists such as Donna Haraway (Haraway, 1988) or Alison Wylie (Wylie, 2003) believe that knowledge, being human knowledge, is based on human perspective. Similarly, Marxist theorists posit that scientific knowledge focuses on the human character of knowledge and is contingent upon issues of gender and race as well as social position (Lektorsky, 1977). Value-neutral theories continue to be propagated based on evidence put forward by researchers and philosophers of science (Levi, 1960), yet we know that the meaning of evidence in and of itself has changed over the ensuing periods. Within the 20th century the debate over what is evidence has been central. Several ideas of evidence as being equivalent to proof or fact, or, more importantly, considered as knowledge, have been set forward. Yet, what creates this evidence, its foundational ground, remains debatable (Djulbegovic et al., 2009).

During the early part of the 20th century, Bertrand Russell advanced the view that “sense data,” objects that we are directly perceptually aware of or items within one’s present consciousness, was mind dependent and could be used to “construct relevant objects of knowledge” (Russell, 1912/1997). However, this then left perceptual information that was indirectly observable out of consideration. For instance, when looking at a table, you can only see the surface and the sides facing you. You can still “see” the table by virtue of seeing only a portion of it.

This leads on to positivist methodological research where the role of the researcher is limited to data collection and interpretation and findings which are typically observable and quantifiable or verifiable. A problem with this view, especially in attempting to prove a hypothesis, is that hypotheses can never truly be proven but only falsified or rejected. Karl Popper, noted philosopher of science, stated that all knowledge and in his view, especially scientific knowledge, is falsifiable. Simply put, just because all swans seen are white in no way logically means that there are no black swans. Knowledge is never complete, it can always be revised or is ultimately fallible (Popper, 2002; 1952). This view, which has been the norm in much research, emphasizes that research is value-free and objective. The underlying belief is that knowledge garnered in this fashion is neutral and based solely on logic regardless of the human or cultural context.

To expand this view, Willard Van Orman Quine defines evidence as that which directly leads from observations with what has been witnessed and is embedded within scientific language (Quine, 1992).

Neither of these are suitable, however, for the inclusion of new information in clinical practice. In “Knowledge and its Limits” Timothy Williamson states that the prerequisites leading to knowledge is what is justified, a true belief, and supports the knowledge base that is being developed. This knowledge base is, ultimately, unanalyzable. And this knowledge, taking it a step beyond the work of the logical positivists, transcends the individual but is, more significantly, community-based and, returning to the work of Quine and others, language-based (Williamson, 2000).

Yet, in recent years, the need for evidence underlying our healthcare systems has increased, as has the publication of evidence-based research such as systematic reviews, random controlled trials and meta-analyses along with less so-called rigorous research protocols.

The story of evidence-based medicine (EBM) is long, though calling it such only dates back to the 1990s.^1^ This was considered to be such an important “break-through” that the New York Times lauded EMB as the idea of the year (Hitt, 2001). Much of what is now talked about in EBM circles was developed within the past two or three decades and comes from the work of epidemiologists and statisticians rather than frontline practitioners. Prior to 1990, medicine was based on the use of evidence or, as the definition states, “grounds of belief,” though often the evidential practices within individual clinics relied upon anecdotal or successful personal experience from specific cases by the practitioner (Howick, 2011).

Looking back to the 1970s, of prime research interest within clinical medicine was a formalized means for patient management. While biomedical knowledge and research was the foundation for clinical application, there was no consistent method for using this information within the day-to-day medical practice. At the time, clinical authority rested with the presiding physician or clinic director. Any questions or concerns were directed toward the very authority who made the initial diagnosis. This view of clinical application began to change. Scientific evidence became an important aspect of care and offered both physicians and patients with the security of relying on what was considered objectively neutral knowledge for decision making. Expert opinion and clinical experience, once considered the basis for clinical decision making, now was placed at or near the bottom of the evidential pyramid (Howick, 2011). Medicine moved from being “the art of medicine” to seeking “best practices.” Diseases are now objectified and unrelated to the subjective sense from the patient.

EBM relies on a reductionist, causal approach to acquire knowledge which is hierarchically ranked with systematic reviews and random controlled trials at the top of the ranking; expert judgment and observational studies carry less significance (Howick, 2011). The goal has been to replace the subjective components of decision making with clinical judgements that are made using purely objective methods. Considered as a pragmatic approach.

To achieve objective knowledge, there is a crucial lack of observational values such as patient preference or cultural needs, that are often the most important component of care according to patients. It follows, based on the intrinsic paradigm, that the research models relying on this reductionist approach reflect the paradigm in which they are found. The assessment of issues around such things as quality of life or patient values are frequently downgraded to secondary importance with the “objective science” carrying most of the weight. And to further complicate the issue, numerous recent studies reveal that the objective knowledge that is so heavily relied upon often have culturally or gendered biases and miss those qualitative components vital for positive patient compliance and outcomes (Hall et al., 2015; Spector and Overholser, 2019). Most studies tended to be biased toward white Western males with, until recently, medical studies typically either ignoring or underrepresenting women (Richardson et al., 2015).

The role of culture and its embedded values including basic assumptions about life, health, politics, societal rituals and mythologies, while easily discerned in many “pre-modern” societies, is felt to have been removed from biomedicine, of which evidence-based medicine is a prime example. Yet, we find that even within differing high technology countries, differences in medical culture exist. Lynn Payer, in her now classic comparative study of medical practice and national culture in the United States, England, France and Germany, reveals that these four western countries, all with equivalent morbidities and life expectancies, have significant differences in medical diagnosis and treatment. What is revealed here is that even with the increase in research-based medicine, cultural influences continue to have an enormous impact (Payer, 1996).

The greatest challenge faced by evidence-based care is to develop practices that are balanced between the best clinical practices encompassing evidence in its various forms with equal consideration of patient values and preferences.

Looking at evidence-based care with its outward appearance of being strictly reductionist, we find that patient care must include those random controlled trials researchers and clinicians so fervently proclaim yet are only a single aspect of what will be integrated patient care. This includes, in the best of circumstance, the consideration of the patient desires, community and cultural aspects that have a direct impact on health outcomes.

What has come to light in recent years has been the paucity of the epistemological foundations of modern evidence-based research. These studies are quite useful for specific types of modalities, specifically drug-related therapies, but are less useful when hoping to understand systems that must take into account greater complexity. Strict links between cause and effect can no longer necessarily be relied upon to clarify treatment results. And the evidence base for the evidence causal hierarchy that has been assumed to be the basis for clinical decision making suddenly becomes suspect when attempting to bridge the gap between efficacy in research and the observations of outcomes in complex systems. Attempts are being made to create a more holistic, non-reductionist set of research protocol suitable for this new plurality of medicine. There has been a failure to understand that evidence can be constructed differently and more appropriately (Barry, 2006) (Figure 1). One such set of protocols was formulated by Wayne B. Jonas as a method to rebalance the evidence hierarchy in a way that orients the methodology used depending on the type of information the provided. Defined as the “House of Evidence,” testing is divided into internal and external validity with each level looking at more or less causal methods. Additionally, he divides each level into testing for effects or testing for use. These are further divided into Mechanism or Meaning at the “lowest level” to Attribution and Association in the middle and Proof compared to General Use at the upper most level (Jonas, 2007).

Beginning in the 1970s, anthropologists began looking at non-biomedical systems as potentially complementary rather than competing (Unschuld, 1976). Alternative health systems challenged allopathic medicine to compete for dominance. Associated with that are the ways and means of understanding how these practices can be integrated into care.

According to Chenyang Li, the Chinese “typically do not see truth as corresponding with objective fact in the world; rather they understand truth more as a way of being…. For them, truth is not carved in stone, and there is no ultimate fixed order in the world” (Li, 2015). In light of this sentiment, the philosophical underpinning of taijiquan reflects the belief that individual experiences reveal a causative principle that can be found within all levels of reality, much of it not observable. Unlike the Western research paradigm with its abbreviated time frame and movement that may be coordinated with breathing, there is a depth that researchers will not see with these limited studies. We may see the outcome but never answer why or how it reaches that point. This then presumes that our understanding of experience is inferential rather than the more Western reductionist model.

Western based researchers continue to attempt to mold taijiquan into a medical model though there is some recognition that there are significant challenges to do so (Wayne and Kaptchuck, 2008a; Wayne and Kaptchuck, 2008b). Interest in traditional medicine was expressed by the World Health Organization, the National Center for Complementary and Integrative Health in the United States and the CAMbrella project supported by the European Union yet also have concerns regarding fraudulent practice, the confirmation of “real” health benefits and levels of professional qualifications. Of particular significance and, as with any area of research, a consensus on research terminology must be established so that researchers can understand one another.

While there are many styles and differing emphases, the fundamental practices of taijiquan are emphasis on slow, mindful movement, deep abdominal breathing and relaxation are a consistent character of the practice. But this is merely the outer aspects of the art. As the practice deepens a sense of a “true essence” is expressed by many practitioners. And this is the issue regarding whether the research actually does represent an authentic practice. The majority of long-term practitioners become aware of the deeper aspects as they delve into these meditative-gymnastic practices. They encompass levels of relaxation and efficient movement that a typical 12-weeks taiji study cannot approach. This is not to denigrate the research nor the effective outcomes but, rather, to clarify the issue of the practice of authentic taijiquan with its combination of mental and physical discipline modeled on specific animal motions, the circulation of qi^2^ and balancing the opposing forces of yin and yang and whether the effects seen can be just as easily created using some other slow, mindful movement (Clarke, 2000).

## Recognition, Integration and Validation of Traditional, Complementary and Alternative Health Care Systems

“Science sits at the root of the efficacy, safety and regulation debate on CAM … science, bolstered by the force of law, has been deployed as a tool of exclusion of nonwestern medical norms… It is for States to embrace factors and paradigms beyond the reductionist framework of Western science or the random controlled trial.”

-Iyioha, 2010

Is it possible to integrate traditional healthcare practices, in this instance the use of taijiquan (and its related practice of qigong) into current Western biomedical systems without the loss of its authentic identity or will it be subsumed into care as simply another therapy, one that has been simplified and separated from the traditional practices found outside of the clinic or research facility? If following the research models currently in use it will, in all likelihood, be the latter.

In other areas of traditional practice vs. biomedical practices challenges are present. We can see tension between the traditional approaches to acupuncture and those that profess to move it into a more Westernized clinical use. The British Medical Acupuncture Society (BMAS), for instance, admittedly claims to be taking a different approach and is re-interpreting the centuries old practices found within traditional acupuncture practices (White et al., 2008). Many GP acupuncturists restrict their practice to chronic pain patients, a much narrower approach than taken by non-medically qualified practitioners. Taking a modern scientific approach, they are attempting to explain the working of this modality in terms that are more aligned with current Western views of anatomy and physiology (White et al., 2008). Additionally, their stance is that the approach traditional acupuncturists take regarding the cause and effect of needling does not accord with current concepts. Though an increasing number of general practitioners use needling and many of the National Health service pain clinics offer it, there remain a significant number of practitioners who have trained and are practicing in a more traditional manner, often with purportedly better response than those who have tried to take a biomedical stand. What is interesting is that even the BMAS recognizes that the experiences of patients undergoing traditional acupuncture treatment are unlike anything experienced and remains inexplicable and that the effects do not appear to follow Western current understanding of how the body functions (White et al., 2008). Joseph Needham has made a point that Eastern and Western science have uniquely different starting points which has led to differing views about physiological function (Needham, 1956, Author’s Note). While no studies are known to date, it would be likely that we would find similar differences arising between traditional taijiquan practice and the Western medical approach.

In addition to questions of inclusion, there are concerns that, in the case of taijiquan, accrediting instructors becomes even more problematic than acupuncture simply because, first, there are numerous styles, second, varying levels of expertize in both the practice and skill of the instructor and finally, how to assess the understanding of the internal aspects of the practice. This last aspect takes years to acquire and “feel.” As we will see, the research remains rather equivocal about the effects of taiji using a Western paradigm.

So, how is it possible to assess the skill and internalization of the practice?

Moreover, there needs to be a mechanism that recognizes the professional group with the authority to set standards for this practice. As with other regulated activities, a system would be needed that both regulates the education of producers, in this case, experts in teaching taijiquan, and also regulates the production by producers, the teaching of taijiquan. This dual aspect of education and regulation is found in other areas where the practitioner is required to be registered such as medicine, dentistry or chiropractic. But in each case, the basic criteria for the profession are known and the education has been formalized. There are also examinations and on-going post-education requirements (Evetts, 2006).

Within the United Kingdom there are several organizations and schools that teach taijiquan but there is no one set of consistent criteria. Attempts have been and are being made to develop a taiji teaching profession within the gym and leisure industry (CIMSPA, 2020). Whether this is possible and if, in doing so, will this alter the art being taught to further match the biomedical model remains to be seen. There has been significant research into how professions develop. What is consistently seen is that the professional associations typically identify, carve out and protect an area of exclusive competency (Saks, 2003). Once this occurs, States grant autonomy and self-regulation leading to licensing. The net effect of this is the standardization of the information being presented, though as mentioned, not necessarily a standard practice. We see this in medical education throughout the biomedical world, though differences do exist (Payer, 1996).

Looking at this from the standpoint of taijiquan, which has several major schools and numerous variants, the systematic body of knowledge comes from the Chinese cultural foundation, yet how it is expressed is highly varied. Expertize certainly is seen within the current taiji teaching community but there is no mechanism for ascertaining with certainty that instructors have a basic minimum of knowledge and understanding of taiji. Validation and accreditation would, in all probability reduce this lack of standardization, especially as the accreditation process stems from a central body responsible for the process.

In the case of taiji practitioners and given the political climate, they could find themselves as merely serving the medical community with the loss of autonomy and an increasing medicalization of both the research and practice of this unique art within the medical setting. What is needed is a new paradigm that would reveal the emergent properties of the consistent practice of taijiquan. Obviously, not an easy proposition, not a short-term fix. In the United Kingdom, the House of Lords Parliament Select Committee on Science and Technology (Wilkinson, 2002) recommended that studies of complementary and alternative medicine, of which taijiquan would be considered, should focus on efficacy before investigating mechanism.

If efficacy testing was based on the wrong mechanism, however, the test may fail to demonstrate value leading to the false conclusion that the treatment does not work (Hyland, 2000). Developing the appropriate testing procedures is crucial.

## *Taijiquan* in the Western Research Context

Taijiquan originated as a martial art. While its definitive origins remain obscure and are rather contentious, several lineages based on specific family styles have grown and changed over the decades. In the West, the art is generally understood as a series of gentle, slow movements with low impact often seen as a movement exercise for seniors or as a health practice to integrate mind/body and create and maintain a sense of well-being and inner peace. The art is a complex, multicomponent mind-body therapy.

One of the more obvious aspects of the practice taiji, such as weight shift, balance and its effects on balance improvement and falls prevention, was the earliest therapeutic aspect that Western research examined. Wolf (Wolf et al., 1996) performed some of the initial research in 1996 looking at the effects of regular taiji practice when compared to computerized balance training in the frail elderly. This 15-weeks, random controlled trial examined the effects of an unspecified taijiquan style on falls reduction in the frail elderly (70+ years of age). The study, being one of the earliest looking at the possible benefits of taiji practice, compared learning several unspecified movements with computerized balance training and, as a third arm, a psychosocial education component. While opening the door for further investigation, this study was greatly flawed from a Western perspective let alone an investigation of the deeper aspects of taijiquan practice, especially since no information was presented regarding the style, the specific of moves taught, why they were selected, and, most importantly, the comparison was not between similar types of movement but rather, quite different methods used to enhance balance and prevent falls.

Falls prevention, still a major health concern with over 37.3 million annual falls world-wide severe enough to require medical assistance (WHO, 2007), became the prime focus for taiji research for the next few years. It was recognized at that time that teaching an entire taiji set of moves was not truly feasible. Researchers started developing protocols that incorporated simplified sets of taiji “exercises” and possibly a few of the accessory warm-up movements. This allowed patients to learn the movements in a brief period of time. The idea was that these selected movements offered the participants the opportunity to experience taiji at a basic level and thereby, develop an understanding of the deeper health supporting principles.

A more recent study investigating balance and falls by Voukelatos et al. (2007), provided once weekly community-based classes for 16 weeks with a follow-up 24 weeks later. In this instance participant were taught either Sun or Yang styles though no specific differentiation was made. While differing styles were used, they were not compared, but simply relied upon as a basis. Once again, there was no indication of which movements had been selected nor why. Additionally, there is no mention of practice outside of the weekly group class so knowledge regarding dose was not established.

Though both studies had positive, if somewhat equivocal results, in neither case were the participants provided with the instruction nor time to learn a complete form. These trials are considered to be, in essence, non-inferiority trials. They were designed to detect whether the intervention, in this case an abbreviated series of movements taken from more established forms, was at least equal to other forms of treatment. Of course, there are other issues at hand such as time spent practicing outside of the controlled environment, co-morbidities, experience of the instructors, dose, etc.

Over the succeeding decades, the research evolved to include different clinical conditions and groups of people. By the late 1990s research had expanded to include acute and chronic heart failure (Guo et al., 2017; Ren et al., 2017), neuropathy (Hermanns et al., 2018), musculoskeletal system (Qin et al., 2005), diabetes (Lee et al., 2014)-and psychological conditions such as anxiety and depression (Kong et al., 2019). The number of studies and the speed with which the research was produced was becoming exponential with the vast number of studies appearing within the last 5 years.

In reviewing a select few studies published in the peer-reviewed literature we find similar difficulties as found with those examining the effects on balance.

For example, the paper by Ren et al. (2017) “The Effects of Tai Chi Training in Patients with Heart Failure: A Systematic Review and Meta-analysis” we find research into another significant health problem with both high morbidity and mortality. Discovering a means to control this problem, given that in the United States alone there are nearly one million cases/year (Benjamin et al., 2017), would be both life-saving and cost-effective. In this paper the authors reviewed a series of published studies in both English and Chinese with the selection criteria of patients suffering heart failure and using any form of taiji as part of their intervention strategy. The control groups were varied and spread across the gamut of “usual care” including pharmacologic therapy, diet, exercise and education or combinations of these. The final selection found 11 suitable randomly controlled studies for inclusion. A limitation of this systematic study was the heterogeneity of the methodology, duration, style and frequency of training. Once again, as we saw with the falls studies, time was limited to 12–16 weeks.^3^ Achieving any true understanding of the art was not likely and could only be assessed if follow-up extended beyond the study timeframe.

Similarly, a meta-analysis by Guo et al. (2017) found that patients with chronic heart failure appeared to improve using a standard 6-min walking distance test. Again, the studies found some possible benefits, though the limitations were significant with regard to poor design, risk of bias and the heterogeneity of the studies. As in previous studies, comparisons between the abbreviated taiji movements and other exercise types was limited and no complete set was taught. While it is evident that there may be reasons to incorporate these movements, labeling them taiji may be a misnomer.

Hermanns et al. (2018), “Impact of Tai Chi on Peripheral Neuropathy Revisited: A Mixed-Methods Study,” looked at the effects of taiji on altered sensation of the limbs due to a number of ill health causes including diabetes, infection or other metabolic conditions. This 12-weeks study reviewed the effects of weekly practice on muscle strength, mobility and balance. Discussions centered on the qualitative component of practice and included questions about well-being, quality of life, etc. In each instance, the participants improved, yet there was no comparison of taiji with other types of movement therapies. As with most studies, the limitations were the small number of participants and the length of time of the study (Hermanns et al., 2018).

“Beneficial Effects of Regular Tai Chi Exercise on Musculoskeletal System” evaluated the of regular practice on bone mineral density. Once again, we find that the study did not compare taiji with any other form of weight bearing exercise. The question remains, does this make taiji unique or will any form of weight-bearing exercise suffice (Qin et al., 2005)? And how has (or does) the limited number of moves taught provide any longer-term benefits?

The paper by Lee et al. (2014), “A systematic review and meta-analysis of taiji for treating type 2 diabetes.” looked at a series of studies of the effects of taiji on diabetes over a period of 10 years. In this instance, several of the reviewed studies compared taiji with walking, dancing or conventional exercise. Another subset compared taiji to standard anti-diabetic medication. These studies, in virtually all instances, did not find taiji to be better than other therapies, in the case of medication, the taiji fared worse (Lee et al., 2014). And again, no effort was made to either teach a complete taijiquan form nor compensate for the heterogeneity of the styles, times taught and nor teachers experience.

Finally, there have also been a significant number of studies using taiji to attempt to alleviate psychological conditions such as anxiety and depression.

“Treating Depression with Tai Chi: State of the Art and Future Perspectives” by Kong et al. (2019) is was one of the few papers that recognizes that teaching a full taiji set required supervision with an experienced teacher. The authors chose not to rely on experienced instructors but rather purposely used a simplified protocol. The scores on the depression scales were improved yet, with the exception of a single study where both taiji and yoga were practiced, there was no comparison between other movement therapies. They do suggest that the core components of mind-body interventions would, potentially, all lead to similar symptomatic improvements including attentional control, emotional regulation and self-awareness and that these therapies can effectively normalize depressive patients cognition but they do not relate this specifically to taiji.

What these studies reveal is that in the majority of instances, based on the Western model, taiji did not really stand up to the expected miraculous effects frequently touted, at least within the timeframes typically used in the research protocols.

These studies appear in an array of medical research journals. The information has filtered into general public awareness and has been reported as the positive effects of taijiquan. Yet, when reviewing the studies, it is consistently found that the length of time in addition to the abbreviated number of movements, leads one to suspect that though the movements used where taken from taiji they were not attempting to study the essence of what make the art unique but rather separate aspects that could be effective in treating specific health conditions. It is not taijiquan that is being taught and researched but rather a series of movements often associated with relaxation and deep breathing. And rarely is there a comparison between taiji and some other set of movements or exercises that are performed in a slow, smooth and relaxed fashion (Jimenez-Martin et al., 2016).

Given the general positive results, it is important to stress that this work is quite significant and important. There is no intent to denigrate the research nor the outcomes. This research offers hope for the inclusion of these exercises into a variety of care settings, however, the question is, are the participants really learning the art of taijiquan or is this simply a set of exercises disconnected or even unrelated to their purported origins? Would any set of movements that includes deep breathing and relaxation suffice? Or is there something unique about the practice and movement? Can the research and practice of taiji be integrated while remaining true to its origins? These questions remain unanswered. The debates are on-going and the theoretical understanding of illness, treatment, prevention need to be addressed.

## Conclusion

Relying on evidence certainly is an important aspect of what can and should be included in care but that returns to the question of what makes the evidence? Whether or not this can be resolved remains within the bounds of the philosophy of knowledge, but we need to be aware that there are contrasting and often opposing points of view. We see this even when examining strictly Western evidence and research.

Taijiquan, as other alternative and complementary practices, has the potential to contribute novel therapeutic and diagnostic modalities to biomedicine (Kidd, 2013) but how we get that information is crucial to fully understanding the mechanisms leading to safety and efficacy and, ultimately, validation and accreditation.

Sitting with the concept of reliable evidence, especially when looking at validation and accreditation, it is apparent that in order for taijiquan to be considered a part of the current biomedical pantheon, how it is researched and prescribed may need to be re-assessed.

The research models currently found within the biomedical health care systems and relying on the concepts of evidence based medicine have proven to be a poor fit for traditional, complementary and alternative health care. In the case of taijiquan, current research protocols, with its limiting timeframe and small number of movements, and an understanding of dose requirements, ignore the potential emergent and highly beneficial properties found in taijiquan with both longer term practice and on-going internal development. And with taijiquan, like similar “traditional” therapies, we find that the whole is greater than the parts. The paradigms that these therapies are modeled on do not fit Western medical concepts and have, for the most part, not been given the necessary depth of study. Can these practices be integrated into current biomedicine? In all probability, only if the medical paradigm shifts to be more inclusive. Any new evidence would need to be integrated within the entirety of the belief system and reframe not just the questions but how we ask those questions. The tension that exists between the safe public use of these therapies, government accreditation and healthcare professions accepting these practices and practitioners is crucial to their use and growth. Biomedicine is solidly scientific, even infallible at least until some life changing event occurs to alter that. At this point in time, this faith in science as the mediator of reality means that self-reflection is limited within evidence-based practices (Montgomery, 2012). New views on how we research and practice need to be developed and framed and include the realization that many practices are not capable of being reduced to minimal common denominators. The shear complexity of the human experience dictates that we re-examine our understanding of the very foundation of that experience.

## Footnotes

1 James Lind is given credit for the first “drug” trial. In 1747 he divided a group of 12 British sailors each suffering from scurvy into six pairs; two were given a quart of cider to drink daily, two a small amount of acid added to their drink, two had nutmeg added to their food, another pair received daily glasses of saltwater and the final pair, oranges to eat and lemons to suck on. The outcome, now classically known, was that the sailors eating the oranges survived while the others died from the ultimate liver failure of scurvy. Acceptance was slow, however, it took another 40 years before the British navy added limes to the diet of their sailors, hence the name “limeys.”

2 Qi (also chi)- Within Chinese culture the vital force that animates a living being. This is said to flow through the body following specific pathways, the meridians. According to Chinese medicine, its unimpeded, smooth flow is what allows one to maintain good health. Taijiquan and the practice of breath work, qigong, are practices that can help with the harmonious flow of qi.

3 A single study extended for 24 weeks but participants were only expected to actively practice for 30 min or less each week.
